# Human stefin B normal and patho-physiological role: molecular and cellular aspects of amyloid-type aggregation of certain EPM1 mutants

**DOI:** 10.3389/fnmol.2012.00088

**Published:** 2012-08-24

**Authors:** Mira Polajnar, Slavko Čeru, Nataša Kopitar-Jerala, Eva Žerovnik

**Affiliations:** ^1^Department of Biochemistry and Molecular and Structural Biology, Jožef Stefan InstituteLjubljana, Slovenia; ^2^IPS Research Group, Jožef Stefan International Postgraduate School (IPS)Ljubljana, Slovenia; ^3^EN-FIST Centre of ExcellenceLjubljana, Slovenia

**Keywords:** epilepsy syndrome, EPM1, cystatin B, protein aggregation, amyloid

## Abstract

Epilepsies are characterized by abnormal electrophysiological activity of the brain. Among various types of inherited epilepsies different epilepsy syndromes, among them progressive myoclonus epilepsies with features of ataxia and neurodegeneration, are counted. The progressive myoclonus epilepsy of type 1 (EPM1), also known as Unverricht-Lundborg disease presents with features of cerebellar atrophy and increased oxidative stress. It has been found that EPM1 is caused by mutations in human cystatin B gene (human stefin B). We first describe the role of protein aggregation in other neurodegenerative conditions. Protein aggregates appear intraneurally but are also excreted, such as is the case with senile plaques of amyloid-β (Aβ) that accumulate in the brain parenchyma and vessel walls. A common characteristic of such diseases is the change of the protein conformation toward β secondary structure that accounts for the strong tendency of such proteins to aggregate and form amyloid fibrils. Second, we describe the patho-physiology of EPM1 and the normal and aberrant roles of stefin B in a mouse model of the disease. Furthermore, we discuss how the increased protein aggregation observed with some of the mutants of human stefin B may relate to the neurodegeneration that occurs in rare EPM1 patients. Our hypothesis (Ceru et al., 2005) states that some of the EPM1 mutants of human stefin B may undergo aggregation in neural cells, thus gaining additional toxic function (apart from loss of normal function). Our *in vitro* experiments thus far have confirmed that four mutants undergo increased aggregation relative to the wild-type protein. It has been shown that the R68X mutant forms amyloid-fibrils very rapidly, even at neutral pH and forms perinuclear inclusions, whereas the G4R mutant exhibits a prolonged lag phase, during which the toxic prefibrillar aggregates accumulate and are scattered more diffusely over the cytoplasm. Initial experiments on the G50E and Q71P missense EPM1 mutants are described.

## Introduction

Epilepsy is a chronic neurological disorder. An epileptic seizure is a transient phenomenon, due to excessive neuronal activity. Classification of different epilepsies is difficult and constantly changing but a rough classification divides them into two major groups of syndromes. The first is a symptomatic epilepsy syndrome in which the epileptic seizures are the result of one or more lesions in the brain. The second group comprises idiopathic epilepsy syndrome, with no underlying structural brain lesions or other neurological signs or symptoms. This latter is presumed to be age and genetic dependent, the genetic epilepsies account for only about 1% of all epilepsies but usually present with severe symptoms and have a poor outcome. The most commonly mutated genes in genetic epilepsies are those coding for protein subunits of voltage-gated (e.g., sodium-gated or calcium-gated) or ligand-gated ion channels (Meisler and Kearney, 2005). These mutations can cause impaired excitability of inhibitory GABAergic interneurons (Rhodes et al., 2004) or hyper excitability of firing neurons (Escayg et al., 2000) which, in either case, disrupts currents in the brain. During excessive activation, glutamate, an excitatory neurotransmitter, causes excessive calcium release that acts on N-methyl-D-aspartate (NMDA) receptors. These are present in large quantities in the hippocampus and thus extremely vulnerable to epileptic seizures and neurodegeneration.

However, mutations other than channel-related ones have been linked to epilepsies. Progressive myoclonic epilepsies (PMEs) are a group of genetic generalized epilepsies with symptoms such as myoclonic and tonic-clonic seizures, dementia and progressive neurodegeneration of gray matter [for reviews see (Shahwan et al., 2005; Ramachandran et al., 2009)]. PMEs can be divided pathogenetically into two groups: non lysosome-related, such as Lafora disease, and lysosome-related, such as Unverricht-Lundborg disease progressive myoclonus epilepsy of type one (EPM1), the most common of all PMEs and the subject of our research. The underlying cause of the appearance of EPM1 is a mutant encoding a cystatin B (stefin B) gene. We later describe the EPM1 mutants, which are aggregation prone *in vitro* and in cells. The hypothesis that this would correlate with more severe cases of EPM1 pathology awaits further confirmation with patient samples.

Protein aggregation to amyloid fibrils is a generic property of proteins (Dobson, 2002), as it has been shown that most proteins, even some α-helical proteins such as myoglobin, can form amyloid-like fibrils. The parts with α helical structure must undergo an α to β transition and the β strands then associate into a fibrillar structure, winding into a β-sheet as protofilaments. Fibril formation has also been observed for proteins which are natively unfolded (intrinsically disordered) (Uversky, 2008) or are predominantly in the β sheet conformation; some of these fold via an α helical intermediate. Different proteins can follow different pathways to amyloid fibrils, depending on the structural class (Zerovnik, 2002; Zerovnik et al., 2011).

Amyloid fibril formation is a process at the core of neurodegenerative diseases, from Alzheimer's disease (AD) and Parkinson's disease (PD) to various other dementias such as Huntington's disease (HD) and amyotrophic lateral sclerosis, but also prion-related diseases and certain hereditary types of epilepsy. Directly or indirectly, most neurodegenerative diseases as a class of conformational disorders (Zerovnik, 2002; Irvine et al., 2008) are connected to **aberrant protein folding**, accompanied by **protein aggregation**. The aggregation can be caused by a mutation (in inherited cases) or by cellular stress and diminishing clearance systems with age (in sporadic cases).

A hallmark of neurodegeneration diseases is loss of memory (Lesne et al., 2006), which is observed long before the major neurodegenerative changes occur in the brain and is to some extent reversible, and therefore accessible to suitable therapy. It is supposed that the pathology at the cellular level is spread from neural axon to the synapse itself and influences the processes of long term potentiation (LTP) (Walsh et al., 2002; Cleary et al., 2005; Miller et al., 2009). It has been shown that neurodegenerative diseases are accompanied by increased oxidative stress and inflammation caused by glia cells activation.

## Progressive myoclonus epilepsy of type 1 (EPM1) as a neurodegenerative disease

EPM1 or Unverricht-Lundborg disease is a type of progressive myoclonus epilepsy (PME), a group of etiologically and clinically heterogeneous inherited disorders sharing a combination of myoclonus, epilepsy, and progressive neurological deterioration (Berkovic et al., 1986). Symptoms range from progressive myoclonic jerks and frequent tonic-clonic seizures, to progressive decline in cognition. EPM1 occurs mainly in Baltic and Mediterranean regions and the onset of the disorder is between 6 and 18 years of age. Initially, patients are mentally alert, showing liability, depression and, later, a mild decline in intellectual performance is observed. However, the disease can lead to dysarthria, and ataxia in later stages (Kalviainen et al., 2008; Genton, 2010). Pathology results in a marked loss of Purkinje cells in the granular layer of the cerebellum (Eldridge et al., 1983).

EPM1 is inherited in an autosomal recessive manner. Mutations in the gene encoding cystatin B (*CSTB*), (we use the alternative name: stefin B throughout), a cysteine protease inhibitor, are responsible for the primary defect underlying EPM1 (Pennacchio et al., 1996; Lalioti et al., 1997a). The most common change found is the dodecamer repeat expansion in the promoter region (Lalioti et al., 1997b), which leads to reduced mRNA and protein levels. The *CSTB* gene is located in chromosome 21q22.3 and altogether 10 different mutations of this gene have been reported to underlie EPM1, as shown in Table 1 (Joensuu et al., 2008).

**Table 1:** **Mutations in *CSTB* underlying Unverricht-Lundborg disease (EPM1) (Joensuu et al., 2008)**.

**Mutation (nucleotide change)**	**Location of mutation in gene/type**	**Predicted consequence for the protein (amino acid change)**
dodecamer repeat expansion	5′ UTR/expansion	reduced CSTB expression
10G > C	exon 1/missense	G4R
67–1G > C	intron 1/splice site	delV23_K56
149G > A	exon 2/missense	G50E
168 > A	exon 2/splice site	aberrant splicing?
169–2A > G	intron 2/splice site	aberrant splicing?
168 + 1_18del	intron 2/deletion	delV23_K56 p.V57EfsX28
202C > T	exon 3/nonsense	R68X
218_219delTC	exon 3/deletion	L73FSX3
212A > C	exon 3/missense	Q71P

Reproduced from Polajnar et al., 2012.

Since the early symptoms can often be mistaken for more common epilepsy types, for example, juvenile myoclonic epilepsy (JME), it is not currently possible to diagnose EPM1 without a genetic test (De Haan et al., 2004).

### Disease development and progression

Stefin B deficient mice (knock-out – KO mice) serve as a model for EPM1, although they lack some of the symptoms typically observed in human patients. KO mice develop myoclonus at 1 month and ataxia at 6 months of age but no tonic-clonic seizures. Myoclonus occurs only during sleep and no photosensitivity nor spike-wave complexes in the electroencephalogram (EEG) are detected (Pennacchio et al., 1998). The hippocampal slices from KO mice are hyperexcitable and more susceptible to kainate-induced tonic-clonic seizures (Franceschetti et al., 2007). Moreover, mice like human patients, show a progressive decrease in neocortex thickness and loss of inhibitory GABA interneurons (Buzzi et al., 2012).

In a developing rat brain, stefin B protein is present in glial cells such as differentiated oligodendrocytes and astrocytes. Stefin B is not expressed in detectable amounts in the cerebellum, except in Purkinje cells of developing and adult rat brain (Riccio et al., 2005). Similarly, in human cerebellum, stefin B resides in Purkinje and Bergmann glial fibers and in the cerebral cortical neurons of the dentate gyrus of the hippocampus. Purkinje cells are GABAergic inhibitory neurons located in the cerebellar cortex and are particularly prone to damage in stefin B KO mice and EPM1 patients (Eldridge et al., 1983; Pennacchio et al., 1998; Franceschetti et al., 2007). Increased excitation of firing neurons due to imbalance between firing and inhibitory neurons is one of the main general causes predicted to underlie all epilepsies. Furthermore, patients show cerebral atrophy and reduced levels of the grey matter in the motor cortex and thalami. Reduced cortical thickness has also been observed (Koskenkorva et al., 2009). Interestingly, the disease has been linked to obesity (Korja et al., 2010). Although stefin B was suggested to play a protective role against apoptosis (Kopitar-Jerala et al., 2005; Yang et al., 2010), it is still unclear how the reduced protein levels could lead to myoclonus and/or the epileptic phenotype with tonic-clonic light sensitive seizures. It was suggested that primary stefin B deficit could trigger several secondary processes, such as overstimulation of serotoninergic transmission (Vaarmann et al., 2006) or a defect in dopamine transmission (Korja et al., 2007), which would in turn cause the seizure phenotype in EPM1.

It has been reported that stefin B can be found in both the cytosol and the nucleus (Riccio et al., 2005; Ceru et al., 2010b), although some authors (Kaur et al., 2010; Yang et al., 2011) did not observe nuclear staining. In a search for its nuclear function, in an astrocytoma cell line T98G stefin B was found to be expressed in the nucleus as well as in the cytosol. Furthermore, it was shown that stefin B interacts with cathepsin L and histones in the nucleus (Ceru et al., 2010a). In synchronized T98G cells, stefin B co-immunoprecipitated with histones H2A.Z, H2B, and H3, predominantly in the G1 phase of the cell cycle. Stefin B-deficient mouse embryonic fibroblasts entered S phase earlier than wild type mouse embryonic fibroblasts. Increased expression of stefin B in the nucleus delayed cell cycle progression, which was associated with the inhibition of cathepsin L in the nucleus, as judged from the decreased cleavage of the CUX1 transcription factor. It was also shown that cells isolated from stefin B deficient animals were more sensitive to staurosporin induced apoptosis than control cells from the wild type animals (Kopitar-Jerala et al., 2005). Whether nuclear or cytosolic functions of stefin B are associated with increased sensitivity to apoptosis in stefin B deficient cells is not yet clear (Abramov and Duchen, 2005).

In most cases the EPM1 mutants lead to lower expression of stefin B mRNA and protein, whose primary function is inhibition of the cysteine proteases. Loss of function symptoms are observed that are relatively well recapitulated in stefin B KO mice. (Kaur et al., 2010) have shown an increase in cathepsin B and D activities in the brain of stefin B KO mice, previously shown to play a role in apoptosis. Moreover, overexpression of cystatin C in these mice reduces the activity of the cathepsins to normal levels and reduces the clinical symptoms and neuropathologies observed in the stefin B KO mice, including motor coordination disorder, cerebellar atrophy, neuronal loss in the cerebellum and cerebral cortex, and gliosis. This partially explains the pathologies appearing in the KO mice. However, double KO mice of cathepsin B and stefin B still present with some residual apoptosis (Houseweart et al., 2003) in accordance with some loss of function other than protease inhibition.

It is therefore important to clarify the protein's alternative function(s), such as in the multiprotein complex important in the regulation of the cytoskeleton (Di Giaimo et al., 2002) and protection of neurons against oxidative stress (Lehtinen et al., 2009). Chaperone-like functions (Skerget et al., 2010; Taler-Vercic and Zerovnik, 2010) have also been predicted. A neuroprotective function of stefin B was proposed in epilepsy on the basis of increased expression in forebrain neurons following seizure activity in rats (D'Amato et al., 2000). Of note, stefin B binds to histones and indirectly regulates the cell cycle through inhibition of cathepsin L in the nucleus (Ceru et al., 2010a). The possibility that stefin B dimers or oligomers would signal cellular pathology to the nucleus should also be considered and proper experiments conducted. If such alternative functions were lost in EPM1 mutants, the consequences could also lead to neurodegeneration.

The gain in toxic function resulting from protein aggregation was predicted for some missense and stop mutants (Ceru et al., 2005). The cytotoxicity of the stefin B aggregates has been proved and also that the aggregates form intracellularly – as will be described in more detail in the following section. In the case of the EPM1 mutants affecting protein sequence (not the dodecamer repeats in the promoter region), which are prone to aggregate in cells, it is not clear whether the protein's mis-folding and aggregation are responsible for augmenting progression of the disease and neurodegenerative changes or whether it is the lack of the protein's protective function (both as protease inhibitor or some alternative function as noted above) or, a combination of the two.

### Possible involvement of stefin B in innate immunity

Glial cells have several important and protective roles in the brain, providing support and nutrition to neurons, maintaining homeostasis of metabolites and metal ions. They also form myelin, and participate in signal transmission in the central nervous system (CNS). There are two groups of glial cells in the CNS: the macroglia, including astrocytes, oligodendrocytes, and ependymal cells, and the microglia. Microglia are effector cells in the CNS that participate in the innate immune defence by continuously surveying their cellular environment in the brain parenchyma (Hanisch and Kettenmann, 2007).

In chronic neurodegenerative diseases the innate immune response is dominated by microglia (Dheen et al., 2007). It has been shown that excessive reactive gliosis by release of the S100β protein inhibits neurogenesis and leads to neuroinflammation and neurodegeneration (Van Eldik and Wainwright, 2003). However, not only the microglia activation but also systemic CNS inflammation contributes to disease progression, as observed in animal models of acute neurodegeneration (Palin et al., 2008), ischemia (McColl et al., 2008) and AD (Dunn et al., 2005). Namely, CNS inflammation can lead to a dysfunctional uptake of glutamate in astrocytes which causes excitotoxicity (Lobsiger and Cleveland, 2007).

In different inflammatory or ischemic conditions, reactive fibrillary astrocytes produce dense microglial scars that build a physical barrier between damaged and healthy cells. This biological phenomenon appears to be a prevalent adaptation against neurodegeneration. Glial activation has also been suggested in various neurodegenerative diseases such as AD and prion disease (Eikelenboom et al., 2002; Mandrekar-Colucci and Landreth, 2010), PD (Tansey et al., 2007) and epilepsy (Ivens et al., 2007). Microglial activation could in fact be one of the main causes of neuronal death (Herrup, 2010) even though studies on post-mortem human AD brain suggest a lack of effective phagocytosis in the clinical samples. However, in stefin B KO mice it has recently been shown that there is localized glial activation in brain regions with significant neural loss that precedes the appearance of myoclonus, which confirms the pathology of excessive glial activation (Tegelberg et al., 2012). Furthermore, neuronal atrophy in the cerebellum (particularly the Purkinje cells), the cortex and the hippocampus are observed in stefin B deficient mice and EPM1 patients (Eldridge et al., 1983; Pennacchio et al., 1998; Koskenkorva et al., 2009). Abundant gliosis (e.g., production of dense glial scars) appears in these regions (Shannon et al., 2002). Genes involved in increased apoptosis and glial activation become overexpressed (Lieuallen et al., 2001).

In accordance with a neuroprotective role for both cystatins C and stefin B (which can to some extent replace each other), Kaur et al., 2010 have shown that cystatin C overexpression in stefin B deficient mice decreases the level of gliosis (Kaur et al., 2010). As for the innate immunity, cystatin C was shown to increase the levels of inducible NO synthase (iNOS) and enhance IFN-γ-induced activation of cystatin C KO macrophages (Frendeus et al., 2009). Cystatin C was also reported to induce microglial activation and neurotoxicity using a rat neuron-microglial model for PD (Dutta et al., 2012). By now it only has been reported that stefin B is involved in the invertebrate innate immunity response (Lefebvre et al., 2004). On the other hand, cystatin C induced autophagy in a neuronal cell-based model, which also exerts neuroprotective effects (Tizon et al., 2010b).

Furthermore, glial cells of the hippocampus and cerebellum participate in synaptic transmission and regulate the clearance of neurotransmitters from the synaptic cleft. These cells surround the synaptic junctions and control neuronal excitability and the strength of synaptic transmission through release of calcium, other ion-fluxes cell adhesion molecules, and specialized signaling molecules released from synaptic and nonsynaptic regions of the neuron (Fields and Stevens-Graham, 2002). Wang et al. (2012) have recently shown that astrocytes modulate the activity of Ca^2+^-dependent uptake of the extracellular K^+^. Active control of the extracellular K^+^ concentration thus provides astrocytes with a simple yet powerful mechanism for rapid modulation of network activity. It is possible that astrocyte calcium signaling and epilepsy are connected (Carmignoto and Haydon, 2012). In fact, the astrocytic activation that occurs in epilepsy results in reduced buffering of extracellular potassium and glutamate, which is suggested to underlie frequency-dependent neuronal hyperexcitability (David et al., 2009). Gliosis and reactive astrocytes were also recently shown in neuronal ceroid lipofuscinosis, a type of PME (Macauley et al., 2011). Therefore, the role of stefin B in astrocytes certainly deserves more study.

## Protein aggregation as a trigger for neurodegenerative diseases

Neurodegenerative diseases are becoming an increasing problem in the Western world due to the prolonged life span of aging populations. Although presenting with different symptoms, they share a common protein mis-folding that leads to aggregation. Here we present a brief overview of the status of our knowledge of protein aggregation in connection with neurodegenerative disease. We then describe, in more detail, aggregation observed in mammalian cell lines of human stefin B, the protein affected in EPM1.

AD is marked by senile plaques, which are extracellular inclusions of predominantly amyloid-β (Aβ) peptide derived from APP (amyloid precursor protein). APP is a transmembrane protein of approximately 700 amino acid residues, which has important prosurvival functions in the cell. It is normally processed by membrane proteolysis (by enzymes called secretases) into an Aβ (1–40) peptide. The more hydrophobic Aβ (1–42) peptide is produced in a higher proportion in some familial cases and also forms oligomeric species and extracellular amyloid plaques. In addition, tau protein becomes hyperphosphorylated and forms intracellular neurofibrillary tangles (Querfurth and Laferla, 2010).

PD is caused by selective and progressive degeneration of pigmented dopaminergic (DA) neurons in the substancia nigra pars compacta (SNpc). One important feature of PD is the presence of eosinophilic, cytoplasmic inclusions of fibrillar aggregates of misfolded protein, termed Lewy bodies, which appear in affected brain areas. The exact composition of Lewy bodies is unknown, except for (ubiquinated) α-synuclein and proteins associated with the genetic forms of PD such as parkin and synphilin (Bossy-Wetzel et al., 2004). These and other proteins linked to PD (e.g., redox-regulated chaperone DJ-1, leucine-rich repeat kinase 2 LRRK2, serine/threonine kinase PINK1) provide further insight into the pathogenesis of PD. Currently, the main mechanism underlying selective dopaminergic neuron death appears to be a severe mitochondrial dysfunction (Bueler, 2009).

Mutation encoding an abnormal expansion of polyglutamine repeats in a protein called huntingtin is the underlying cause of HD. Disease severity depends on the length of the polyQ stretch. Repeats longer than 37 are clearly linked to HD. The expanded polyQ stretch of the mutated huntingtin induces conformational change to a cross-β structure, resulting in aggregates that are found in dendrites and nuclei of the affected neurons. The aggregates, especially soluble oligomeric forms, are thought to confer a gain in toxic function via transcriptional deregulation, binding and sequestering of selective transcription factors, protein aggregation, causing excitotoxicity (Friedlander, 2003; Bossy-Wetzel et al., 2008). Symptoms of HD include movement disorder (Huntington's chorea), cognitive dysfunction, and psychiatric symptoms such as depression and psychosis. This results from selective loss of the long projection GABAergic neurons, known as medium spiny neurons, which are responsible for control of movement both of the body and limbs (Bossy-Wetzel et al., 2008).

Prion diseases (e.g., kuru, fatal familial insomnia) can originate spontaneously or are inherited. The main component of prion pathology is a misfolded, partially protease-resistant “scrapie” conformer (PrP^Sc^) derived from a normal cell surface protein, the cellular prion protein (PrP^C^), which exerts an anti-oxidative function. Other pathological features common to prion diseases and other neurodegenerative disorders are astrocytosis and vacuolization (Sakudo and Ikuta, 2009).

Cellular prion protein (PrP^C^) was found to function as a high affinity surface receptor for soluble Aβ-oligomers in neurons and is therefore a mediator of the Aβ-oligomer induced synaptic dysfunction. This links AD with infective human prion diseases, such as variant Creutzfeldt-Jakob disease (CJD) (Lauren et al., 2009). The infectivity of other amyloid forms connected to PD and HD has also been studied, however, there is no direct evidence of infectious forms of Aβ, tau, huntingtin or α-synuclein (Cushman et al., 2010).

There are more than 25 such amyloid forming proteins. To learn of such proteins and the corresponding diseases see Tables in reviews (Yon, 2002; Polajnar et al., 2012).

### Defence of cells against toxic effects of protein aggregates

Evidence exists that soluble, prefibrillar aggregates are more cytotoxic than mature and insoluble amyloid fibrils (Bucciantini et al., 2002, 2004, 2005; Stefani, 2010). Smaller protein aggregates are transported in a microtubule-dependant manner to the centrosome in the vicinity of the nucleus, and are sequestered into vesicular structures, called aggresomes (Kopito, 2000). The aggresome serves as a storage compartment for intracytoplasmic protein aggregates. One suggestion is that the formation of aggresomes is a protective mechanism that sequesters the toxic species of abnormally folded polypeptides (Corboy et al., 2005).

There are several mechanisms in cells that help prevent toxicity of protein aggregates. These comprise various chaperone proteins, the ubiquitin proteasome system (UPS) and autophagy. The latter often serves as the last line of defence.

While UPS malfunctioning has already been linked to neurodegenerative disorders, important links between autophagy and neurodegeneration have also become evident (Ventruti and Cuervo, 2007). Studies have revealed cross-talk between UPS and autophagy, suggesting a coordinated and complementary interaction between these two degradation systems, especially in cases of cellular stress (Nedelsky et al., 2008). Autophagy is a degradative and recycling process of many cellular components ranging from long-lived proteins to whole organelles. It increases during starvation, oxidative stress and hypoxia, i.e., pathological conditions that cause cellular stress. Macroautophagy is the most common type of autophagy. The turnover of cell debris occurs via double-membrane vesicles termed autophagosomes in which it is degraded by lysosomal proteases in the low pH environment. Cytosolic proteins, as well as misfolded secretory proteins and some membrane proteins exported from the endoplasmic reticulum to the cytosol by retrograde transport, are normally degraded by UPS, however, autophagy is the predominant way of aggresomes clearance (Chin et al., 2010).

Autophagy can be impaired in many neurodegenerative disorders [for review (Wong and Cuervo, 2010)]. We recently proposed that autophagy may also be impaired in progressive myoclonus epilepsies, including EPM1 and EPM2 (Polajnar and Zerovnik, 2011). Impairment of autophagy could be due to dysfunction in the lysosomal pathway and/or the susceptibility of the lysosome to oxidative stress that can result from excessive protein aggregation, mitochondrial dysfunction or other cellular defects. Furthermore, amyloid forming proteins in their prefibrillar and oligomeric forms can disrupt membrane integrity and even make channels (pores) which resemble those of bacterial pore-forming toxins [for review see (Butterfield and Lashuel, 2010)]. One of the biggest sources of reactive oxygen species (ROS) are (intact) mitochondria. Production of ROS can be significantly increased in various neurodegenerative disorders due to mitochondrial defects in the respiratory and energy chains (Lim et al., 2008; Vila and Perier, 2008; Rhein et al., 2009). Indeed, it has been observed that the high level of H_2_O_2_ that originates from mitochondria of starved cells acts as a signal to initiate autophagy (Scherz-Shouval and Elazar, 2007). Dysfunctional mitochondria can finally collapse and proapoptotic molecules are released which leads to cell death (Halestrap, 2009).

Recently, cystatin C was shown to induce autophagy via mTOR inhibition, which is pro-survival for the cell under stress conditions (Tizon et al., 2010a). Cystatin C has diverse functions, the best known being inhibition of cysteine proteases such as cathepsins, papain (Brzin et al., 1984; Turk and Bode, 1991) and legumain (Alvarez-Fernandez et al., 1999). Furthermore, cystatin C was shown to interact with Aβ and to inhibit formation *in vitro* of Aβ fibrils (Sastre et al., 2004) and oligomers (Selenica et al., 2007; Tizon et al., 2010a). Cystatin C bound to the soluble Aβ in the brains of Aβ amyloid depositing transgenic mice and inhibited the aggregates and deposites of Aβ plaques in the brain (Kaeser et al., 2007; Mi et al., 2007). Cystatin C could exert a neuroprotective function by either preventing cell death, or promoting cell survival and neurogenesis.

Stefin B is part of the same cystatin superfamily as cystatin C and inhibits cathepsins B, C, H, K, L and S (Turk et al., 1986; Lenarcic et al., 1996; Dahl et al., 2001). Similar to cystatin C, stefin B inhibits Aβ fibril growth, however, this binding is dependent on its oligomeric state (Skerget et al., 2010), with the tetramer showing the highest affinity for Aβ. Oligomers of stefin B have been observed in cells (Cipollini et al., 2008). It also has been shown that the endogenous protein forms smaller, occasional aggregates, which become more abundant upon proteasome inhibition or in the presence of mutations (e.g., R68X) (Ceru et al., 2010b). Recently, neuronal cytoplasmic and intranuclear inclusions containing the lysosomal proteins cathepsin B and CD68 and FUS, respectively, were identified in one EPM1 patient (Cohen et al., 2011).

### Human stefin B EPM1 mutants aggregate readily *in vitro*

Stefin B has served as a good model system with which to study amyloid fibril formation and prefibrillar aggregation (Zerovnik, 2002; Anderluh et al., 2005; Rabzelj et al., 2005; Zerovnik et al., 2007). Its sequence, which is prone to oligomerise and form amyloid fibrils, is 52% identical to that of a more stable homologue, stefin A, which is predominantly a monomeric protein and can only be pushed to fibrils under extreme conditions (Jenko et al., 2004). The stability and aggregation propensity of several stefin B mutants and one iso-form (variant bearing Y31) have been studied (Jenko et al., 2004; Kenig et al., 2004; Rabzelj et al., 2005; Kenig et al., 2006; Ceru et al., 2010b). In 2005 we proposed (Ceru et al., 2005) that some of the mutants of human stefin B found in EPM1 patients, which are not active as cysteine protease inhibitors, may be more prone to aggregation than the wild-type protein and that they also probably aggregate inside the mammalian cell, producing additional gain in toxic function (Zerovnik et al., 2002; Anderluh et al., 2005).

Sizes and morphology of the prefibrillar aggregates have been determined and are similar to those of other amyloid forming proteins (Ceru et al., 2008). Furthermore, toxicity studies showed that the prefibrillar oligomers of stefin B are toxic to cells (Ceru et al., 2008; Ceru and Zerovnik, 2008). A consensus has been reached that the mechanism of amyloid cytotoxicity involves membrane perturbation or perforation (Stefani, 2010). As observed for a number of amyloid forming proteins, stefin B binds to acidic phospholipid bilayers (Anderluh et al., 2005; Rabzelj et al., 2008).

Several mutants of stefin B, occurring in EPM1 patients, which bear changes in the coding region of the protein sequence were prepared in *Esherichia coli.* Their inhibitory activity as cysteine protease inhibitors has also been measured. The G4R mutant showed no inhibitory activity at all, while G50E and Q71P mutants showed much lower inhibitory activity than the wild type protein. The fragment R68X, which stays unfolded, was not active, either.

It has been shown *in vitro* that the fragment R68X forms amyloid-fibrils very rapidly, even at neutral pH and under mild conditions, whereas the G4R mutant exhibits a prolonged lag phase, during which prefibrillar aggregates accumulate (Rabzelj et al., 2005). These aggregates and oligomers, when added to the cell medium, produced a toxic effect (Ceru et al., 2008). The isolated higher-order oligomers bound effectively to lipid monolayers and were shown to get internalized to the cell cytoplasm from the medium where they reduced cell viability (Ceru et al., 2008; Ceru and Zerovnik, 2008; Rabzelj et al., 2008).

G50E and Q71P mutants, on the basis of CD and ANS fluorescence spectra, possess secondary and tertiary structures similar to that of the wild type, only they are slightly more unfolded and have exposed hydrophobic surfaces (Polajnar et al., 2011). The R68X fragment is unfolded and transforms to amyloid-like fibrils at neutral pH (Rabzelj et al., 2005). Interestingly, and in distinction from other missense EPM1 mutants, G4R is a properly folded and compact protein of similar stability to the wild type protein but exhibits a four times longer lag phase during fibril formation (Rabzelj et al., 2005).

### Human stefin B EPM1 mutants form aggregates in cells and show cyto-toxicity

Cell culture experiments have confirmed that stefin B is prone to form oligomers and aggregates in cells (Cipollini et al., 2008). The endogenous protein forms small oligomers while the wild type stefin B and its EPM1 mutants aggregate upon overexpression, the wild type to a much lesser extent (Ceru et al., 2010b). This suggests that the regulation of both expression and oligomerization of stefin B is crucial to the cell and may have a role in neurodegeneration.

**Figure 1:**
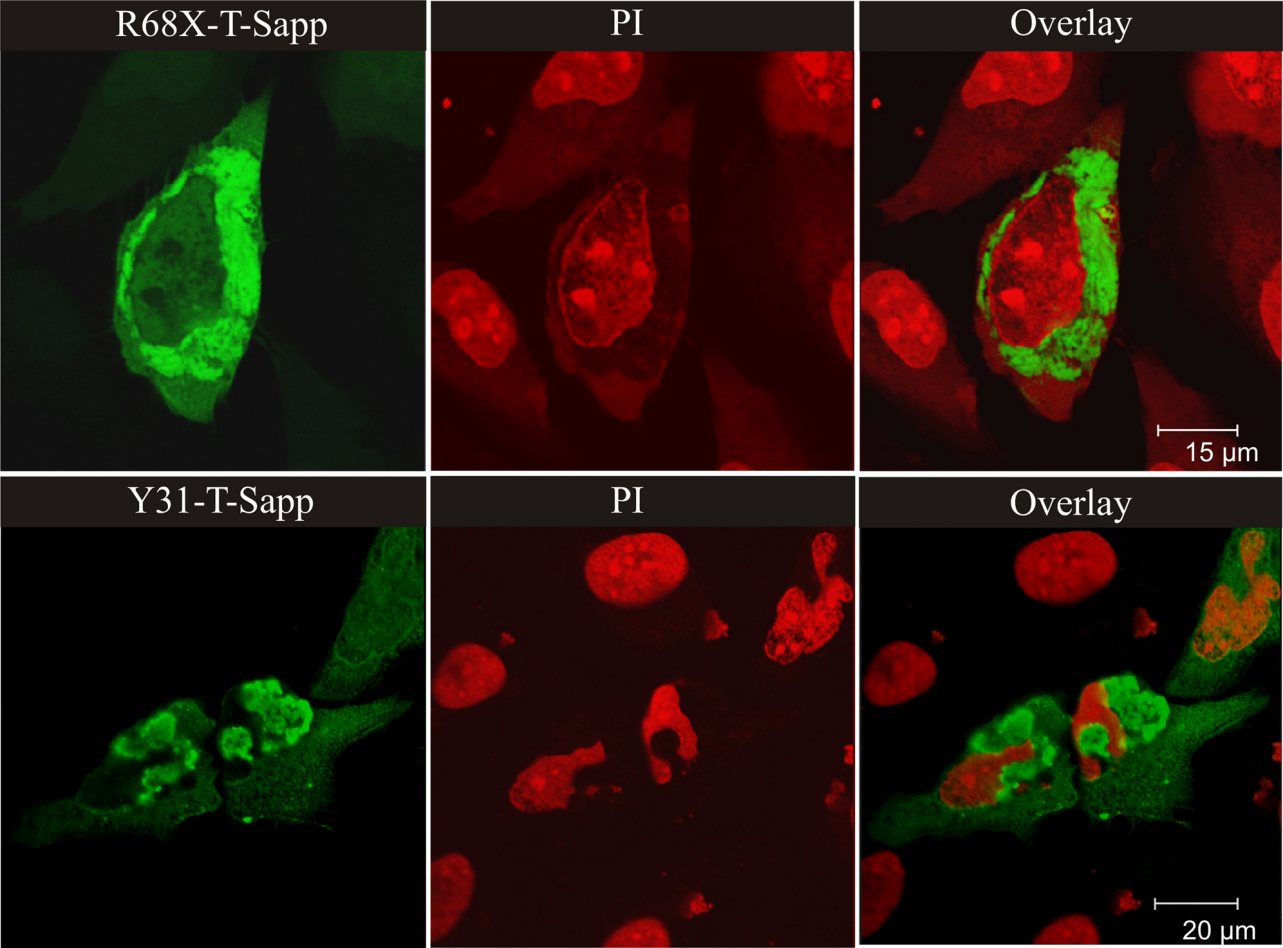
**Perinuclear aggregates of the EPM1 R68X mutant and of the most aggregate prone wild-type variant stefin B with Y31 (both fused with green fluorescent protein T-Sapphire).** The nuclei were labeled with propyl-iodide – PI (red color). The wild type protein does not form substantial aggregates at the same level of expression. For Figure 1, from the journal “Amyloid”, we obtained the copyright by Informa Healthcare via RightsLink.

As compared to the wild-type protein, all the missense mutants we have studied formed larger cytosolic, and often also perinuclear, aggregates (see, Figure 1). The aggregates of stefin B mutants have many of the molecular characteristics of aggresomes (Kopito, 2000) as partially demonstrated previously (Ceru et al., 2010b). In contrast to the belief that aggresomes could be cytoprotective (Nedelsky et al., 2008), in our case the cells presenting more abundant aggregates were less viable. The partial co-localization of stefin B R68X aggregates with LC3 indicates that the aggresomes could be targeted for autophagic clearance (Ceru et al., 2010b).

The G50E and Q71P stefin B mutants also form large perinuclear aggregates that cause collapse of the cell and fragmentation of the nucleus [Figure not shown, data were presented at FEBS 2011 conference (Polajnar et al., 2011)].

It has been demonstrated (Ceru et al., 2008) that the R68X fragment forms aggresome-like structures at the nucleus which are cytotoxic, whereas the G4R mutant and the wild type protein form smaller aggregates under conditions of over-expression or proteasome inhibition. Consistent with this, patients bearing one allele of R68X and the other of the usual dodecamer repeat in the cystatin B gene, show more severe pathology and clinical symptoms (Koskenkorva et al., 2011). These findings raise the question as to whether the oligomers/aggregates are cytotoxic or whether the inclusions are instead protective, as suggested by some (Kopito, 2000).

Why no-one has reported that EPM1 would be a conformational disorder, bearing protein aggregates of stefin B intracellularly? Only rare studies of samples from human patients with EPM1 can be found in the literature. Also, mutations occurring in the exonic or intronic region of cystatin B gene account for only 10% of the EPM1 patients. Protein aggregates have been discovered recently in the brain of one EPM1 patient, however, these aggregates did not contain stefin B (Cohen et al., 2011). This was a patient with a dodecamer expansion in the promoter region which causes decreased expression of stefin B protein. We are not aware of detailed neuropathogical studies performed on patients with additional mutations (especially those in the transcript region). Of note, it was reported that patients with an R68X mutation, which leads to expression of a truncated version of stefin B protein, have more severe myoclonus, drug-resistant tonic-clonic seizures and lower cognitive performance. Some were even reported to be psychotic (Koskenkorva et al., 2011). Our results, albeit obtained from experiments on cell cultures, strongly suggest that stefin B becomes more toxic when it forms aggregates and thus should be considered in future human pathological studies.

## Conclusions

*In vitro* experiments have confirmed that most of the exonic EPM1 mutants of human stefin B are misfolded proteins, either completely unfolded such as R68X, or partially unfolded such as G50E and Q71P, exposing increased amounts of hydrophobic patches. These structural characteristics indicate instability and lead to protein aggregation. An exception is the G4R mutant which possesses completely folded secondary and tertiary structures but still loses its protease inhibitor function. When over-expressed, even this mutant can form diffuse aggregates while the other EPM1 mutants, G50E, Q71P and R68X, form large perinuclear aggregates that sometimes extend over the whole cytoplasm. The aggregates are toxic (Ceru et al., 2010b) which could arise through a different mechanism, e.g., pore formation in cellular or plasma membranes, increased oxidative stress or autophagy disruption. Data confirming such a mechanism of toxicity are being collected.

We argue that, regardless of the fact that in EPM1 patients no intracellular inclusions of stefin B have been observed thus far (but a conference report by Cantor that mast and lung cells have vesicular inclusions of unknown composition—Eva Žerovnik personal communication), the aggregation of the EPM1 mutants R68X, G50E, Q71P and, to a smaller extent G4R, could modulate the phenotype of the disease, by a gain in toxic function.

There can be several reasons why no inclusion bodies have been observed in patients. Either, the rare cases of these mutants (only one allele of about 10% of EPM1 cases may bear a gene mutation to code changes in the sequence such as R68X, G50E and P71Q) might have been overlooked, or lack of stefin B rather contributes to aggregate formation of other proteins—if it acted as a chaperone (Taler-Vercic and Zerovnik, 2010).

### Conflict of interest statement

The authors declare that the research was conducted in the absence of any commercial or financial relationships that could be construed as a potential conflict of interest.
